# Glucose consumption rate-dependent transcriptome profiling of *Escherichia coli* provides insight on performance as microbial factories

**DOI:** 10.1186/s12934-022-01909-y

**Published:** 2022-09-14

**Authors:** Juan Carlos Fragoso-Jiménez, Rosa María Gutierrez-Rios, Noemí Flores, Alfredo Martinez, Alvaro R. Lara, Frank Delvigne, Guillermo Gosset

**Affiliations:** 1 Departamento de Ingeniería Celular y Biocatálisis, Instituto de Biotecnología, Universidad Nacional Autónoma de México, Morelos Cuernavaca, México; 2 Departamento de Procesos y Tecnología, Universidad Autónoma Metropolitana, Ciudad de Mexico, México; 3 Terra Research and Teaching Centre, Microbial Processes and Interactions (MiPI) Gembloux Agro‑Bio Tech, University of Liège, Gembloux, Belgium

**Keywords:** Transcriptome, Glucose transport, Mutant, RNA-seq, Physiology

## Abstract

**Background:**

The modification of glucose import capacity is an engineering strategy that has been shown to improve the characteristics of *Escherichia coli* as a microbial factory. A reduction in glucose import capacity can have a positive effect on production strain performance, however, this is not always the case. In this study, *E. coli* W3110 and a group of four isogenic derivative strains, harboring single or multiple deletions of genes encoding phosphoenolpyruvate:sugar phosphotransferase system (PTS)-dependent transporters as well as non-PTS transporters were characterized by determining their transcriptomic response to reduced glucose import capacity.

**Results:**

These strains were grown in bioreactors with M9 mineral salts medium containing 20 g/L of glucose, where they displayed specific growth rates ranging from 0.67 to 0.27 h^−1^, and specific glucose consumption rates (*qs*) ranging from 1.78 to 0.37 g/g h. RNA-seq analysis revealed a transcriptional response consistent with carbon source limitation among all the mutant strains, involving functions related to transport and metabolism of alternate carbon sources and characterized by a decrease in genes encoding glycolytic enzymes and an increase in gluconeogenic functions. A total of 107 and 185 genes displayed positive and negative correlations with *qs*, respectively. Functions displaying positive correlation included energy generation, amino acid biosynthesis, and sugar import.

**Conclusion:**

Changes in gene expression of *E. coli* strains with impaired glucose import capacity could be correlated with *qs* values and this allowed an inference of the physiological state of each mutant. In strains with lower *qs* values, a gene expression pattern is consistent with energy limitation and entry into the stationary phase. This physiological state could explain why these strains display a lower capacity to produce recombinant protein, even when they show very low rates of acetate production. The comparison of the transcriptomes of the engineered strains employed as microbial factories is an effective approach for identifying favorable phenotypes with the potential to improve the synthesis of biotechnological products.

**Supplementary Information:**

The online version contains supplementary material available at 10.1186/s12934-022-01909-y.

## Introduction

The bacterium *Escherichia coli* is widely used both in academia and industry. The culture media for growing this organism usually contains glucose, since this carbohydrate is relatively inexpensive, and is the preferred carbon and energy source of *E. coli* [1]. When *E. coli* grows under conditions where both glucose and oxygen are non-limiting, it displays high specific glucose consumption (*qs*) and growth rates (*µ*) with concomitant acetic acid synthesis. Acetic acid production has been a widely discussed topic by many authors. The production of acetic acid can be explained as an unbalance between the glucose uptake flux and the rate of conversion of the molecules derived from this sugar to energy and the metabolic products diverting acetyl coenzyme A (acetyl-CoA) from the tricarboxylic acid cycle (TCA) towards acetate. Acetate production is not dependent on oxygen availability, so it can occur under aerobic conditions. At low growth rates, glucose can be completely oxidized to carbon dioxide. However, when the growth rate increases, at certain threshold overflow metabolism is observed. This phenomenon coincides with the highest rate of oxygen consumption, above this value, overflow metabolism is evident. In summary, acetic acid production has been related to the saturation of TCA and respiratory chain capacity, as well as insufficient coenzymes replenishment [2–6]. Since acetate is a toxic compound, its accumulation has a negative effect on *E. coli* growth and productivity [7, 8]. Various approaches have been explored to try to reduce or eliminate acetate accumulation in cultures with *E. coli*. These include adjustments to the fermentation process, as well as strain genetic modifications. The use of various glucose feeding strategies for limiting the accumulation of acetate in the culture medium is an effective strategy to reduce overflow metabolism. Another successful approach is based on genetic modifications that eliminate acetate synthesis pathways or reduce glucose import capacity by deleting genes that encode transporter proteins. These strategies have been proven to be successful in increasing the production of chemicals, plasmid DNA vaccines, and recombinant proteins [9–12].

Glucose transport and phosphorylation in *E. coli* are dependent on the phosphoenolpyruvate:sugar phosphotransferase system (PTS) [13]. Protein IICB^Glc^, encoded by gene *ptsG*, is the glucose-specific PTS component. Under glucose limitation conditions, a high-affinity glucose uptake pathway composed of the outer membrane protein LamB and the MglABC transport system is induced [14]. It is also known that the galactose:H + symporter GalP can import glucose which is afterward phosphorylated by the glucokinase enzyme in an ATP-dependent reaction [15]. It has also been reported that PTS components, such as IICD^Man^ which is normally involved in mannose import, can replace the function of IICB^Glc^ for glucose import [11, 16, 17].

In *E. coli*, internal metabolic fluxes, as well as the phosphorylation state of PTS proteins, allows this organism to sense if glucose is abundant or limited in the culture medium and if other carbon sources are present. Carbon catabolite repression (CRR) can be defined as the hierarchical consumption of carbon sources that allow the highest growth rate [18–20]. As we have mentioned before, glucose is the preferred carbon and energy source of *E. coli*. This carbohydrate is simultaneously transported and phosphorylated by the glucose-specific PTS components. One of these components is the protein PTS EIIA^Glc^ which transfers a high-energy phosphate group from protein HPr to the glucose-specific component IICB^Glc^. Protein EIIA^Glc^ is non-phosphorylated when glucose is actively transported and metabolized by the cell and in this state, it is available to bind to other non-PTS permeases inhibiting the uptake of alternate carbon sources such as lactose. On the contrary, when glucose is absent in the medium, EIIA^Glc^ is mainly phosphorylated, and in this state, it is available to bind to the adenylate cyclase enzyme (CyaA), stimulating its activity, thus increasing the intracellular concentration of cyclic AMP (cAMP)[1, 18, 19]. An increased amount of cAMP and its binding to the cAMP receptor protein (CRP) leads to the expression of catabolite-repressed genes, enabling the utilization of alternative carbon sources [1, 18, 19]. In another example of cellular response to glucose availability, it has recently been reported that hydrogenase 4 subunits HyfBDF can sense two different glucose concentrations (2 or 8 g/L). Depending on the amount of glucose in the medium, hydrogenase 4 can regulate the H + /K + fluxes to maintain proton motive force generation [21].

The physiological response of *E. coli* to nutrient limitation has been studied extensively [14, 22–25]. When glucose concentration is limiting, a hunger state is defined if it causes a reduction in the *µ* to a value below the maximal rate observed when this nutrient is abundant [14]. In contrast, a starvation state is defined by growth cessation because of glucose depletion. The hunger response is characterized by the expression of nutrient scavenging systems, including genes encoding proteins involved in high-affinity glucose uptake such as LamB and MglABC, that replace PTS-dependent glucose import by OmpC and IICB^Glc^ [14]. Knowledge about the global transcriptional response of *E. coli* to glucose limitation has been extended by using technologies such as qPCR, microarrays, and RNA-seq. These studies have enabled a better understanding of the physiological state under specific conditions, providing insight into the roles of cellular components on cell adaptability and survival [23, 26–28]. Among key findings is the elucidation of regulatory components coordinating the response to glucose limitation. The global regulator CRP, bound to its effector cAMP, has been determined to control about one-third of the genes responding to this condition [29]. In addition, growth rate-dependent control, including the alternative sigma factor RpoS and small untranslated RNAs is involved in the carbon source limitation response [27, 30].

Even though much is known about how *E. coli* responds to nutrient limitation conditions, there is growing interest in understanding how glucose-limitation responses would relate to the performance of this organism as a biotechnological production host. It would be useful to characterize *E. coli* strains that are improved cell factories for biotechnological products. In this regard, a set of mutant strains have been generated to display distinct glucose consumption rates, based on the deletion of PTS and non-PTS glucose importers [11, 31, 32]. These strains import glucose at fixed values that cause a growth rate below the maximum. Thus, these mutants can emulate the dilution rates of continuous culture, but without any external process control [11, 12]. When compared to wild-type *E. coli* W3110, some of these mutant strains have displayed increased production of a DNA vaccine and membrane proteins [11, 32]. A recent study compared the capacity for GFP production among a group of six isogenic strains that exhibit distinct *qs* values as a result of the deletion of glucose transporters. *E. coli* W3110 and mutant derivatives displayed *qs* ranging from 1.75 to 0.45 g/g h. It was determined that all mutant strains produced a 4 to 25 higher GFP titer when compared to W3110 [12]. These results can be explained in part by the observed reduction in acetate accumulation in the culture medium. However, the acetate titer and specific rate of production did not correlate with GFP yield or titer [12]. These data strongly suggest that a reduction in *qs* results in modifications of cell physiology that go beyond a reduction in overflow metabolism and these perturbations have an impact on recombinant protein production capacity. Thus, it is of interest to determine the physiological state of these strains to help in understanding the basis for their improved performance. To address these questions, we performed comparative transcriptome analyses.

## Material and methods

### Strains and cultivation conditions

**Fig. 1:**
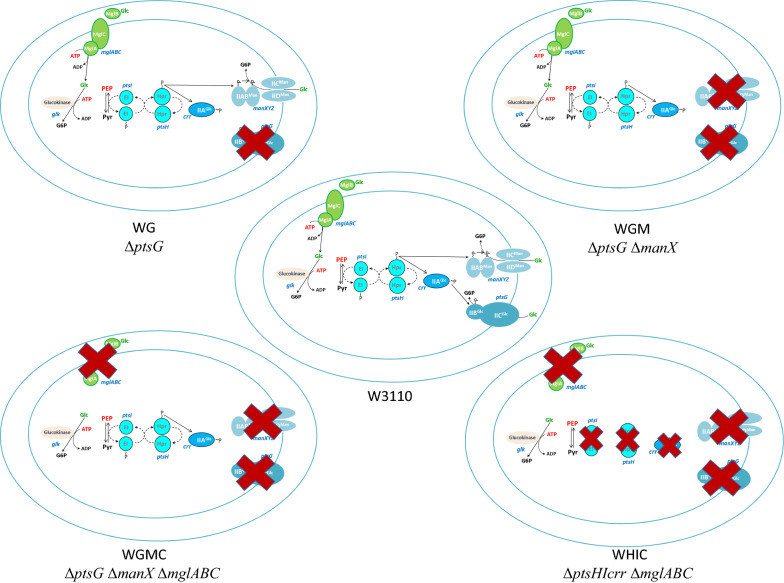
Description of the strains employed in this study. WGΔ*ptsG*::FRT; WGM Δ*ptsG*::FRT, Δ*manX*::FRT; WGMC Δ*ptsG*::FRT, Δ*manX*::FRT, Δ*mglABC*::FRT-Cm-FRT; WHICΔ*ptsHIcrr*::FRT-Cm-FRT Δ*mglABC*::FRT-Cm-FRT

The strains used in this work are described in Table 1 and Fig. 1. The strain *E. coli* W3110 is a derivative of K-12 [33]. Strains WG, WGM, WGMC, and WHIC are derivatives of W3110 [11]. All the strains were cultured in 5 mL Luria–Bertani medium, stored in 40% glycerol, and kept frozen at − 70 °C. These strains were grown in stirred tank bioreactors, using M9 minimum salts medium supplemented with 20 g/L of glucose, and 0.15 mL/L of trace elements solution. The M9 medium contained 6 g/L Na_2_HPO_4_, 3 g/L KH_2_PO_4_,0.5 g/L NaCl, 1 g/L NH_4_Cl, 0.5 g/L MgSO_4_, 0.01 g/L CaCl_2_, 0.01 g/L thiamine hydrochloride. The trace elements solution contained the following: 1.5 g/L Na_2_EDTA · 2H_2_O, 0.45 g/L ZnSO_4_ · 7H_2_O, 0.03 g/L MnCl_2_ · 4H_2_O, 0.1 g/L H_3_BO_3_, 0.04 g/L Na_2_MoO_4_ · 2H_2_O, 0.3 g/L FeSO_4_ · 7H_2_O, and 0.03 g/L CuSO_4_ · 5H_2_O. Glucose and salts solutions were sterilized separately at 121 °C for 20 min. The inoculum for bioreactor cultures started with a single colony placed in a tube with 5 mL of LB medium and incubated at 37 °C, 300 rpm in an orbital shaker. After 8 h, a sample of this culture was used to inoculate a 250 mL shake flask containing 50 mL of M9 medium with 20 g/L glucose starting at an OD 600 nm of 0.1 and incubated at 37 °C, 300 rpm until an OD 600 nm of 2.0 was reached. Then a sample from this culture was used to inoculate sterile bioreactors containing 750 mL of the same medium, starting at an OD 600 nm of 0.1. Batch cultures in stirred tank bioreactor were performed using 1 L autoclavable glass bioreactors (Applikon, The Netherlands). The reactors were equipped with controls for pH, temperature, agitation, and dissolved oxygen. 3% NH_4_OH and H_3_PO_4_ solutions were automatically added to control pH at 7.0. The temperature was maintained at 37 °C, dissolved oxygen was manually maintained above 20% by changing stirring speed, and airflow was set to 1 vvm. Samples were taken periodically for offline analyses. All cultures were performed in triplicate.

**Table 1:** E. coli strains employed in this study

Name	Description	Sources
Strains		
W3110	*E. coli* F- λ—*rph-1 IN(rrnD-rrnE)*1	[33]
WG	W3110 Δ*ptsG*::FRT	[11]
WGM	WG, Δ*manX*::FRT	[11]
WGMC	WGM, Δ*mglABC*::FRT-Cm-FRT	[11]
WHIC	W3110 Δ*ptsHIcrr*::FRT-Cm-FRT Δ*mglABC*::FRT-Cm-FRT	[11]

### RNA extraction, purification, mRNA library construction and RNA-seq

We carried out cultures for each strain under the same conditions as described above. Cultures were started at an OD 600 nm of 0.1 and growth was monitored until each strain reached an OD 600 nm of 1.0. At this point (mid-exponential growth phase), a 50 mL sample of culture medium was taken, placed in an ice bath, immediately added 1 ml of RNA later buffer (Ambion Inc., USA) mixed it, and centrifuged 10 min at 4 °C at 5000 rpm. The pellet was immediately kept frozen at − 70 °C. To perform RNA-seq sequencing, RNA extraction was made using the RNeasy Midi Kit (Qiagen) following the manufacturer’s instructions. RNA extractions and purifications were carried out from three independent cultures for each strain.

The ribosomal ribonucleic acid (rRNA) integrity was determined by the Bioanalyzer 2100 system (Agilent Technologies, Inc., Santa Clara, CA) using an Agilent RNA 6000 Nano chip, RNA Integrity Number (RIN) values were obtained for each RNA sample, and we only considered those with a value higher than 8 to elaborate RNAseq libraries. Subsequently, bacterial rRNA was depleted using Ribo-Zero rRNA Removal Kit for Bacteria (Epicentre™) following the manufacturer’s instructions. RNA was analyzed using the Bioanalyzer 2100 once again. Afterward, RNAseq libraries were constructed using Illumina TruSeq Stranded mRNA sample prep kit (Illumina, Inc., San Diego, CA) from RNA obtained previously, and following instructions of the manufacturer. Briefly, RNA was chemically fragmented by divalent cations to generate 200–600 bp fragments. After that, the cDNA of each fragmented RNA sample was synthesized with retrotranscriptase enzyme using degenerated primers of 6 nucleotides. dsDNA was obtained from cDNA using a Klenow DNA polymerase. Once dsDNA was obtained, both ends of each molecule were repaired to join with Illumina adapters by employing T4 DNA polymerase and T4 DNA ligase. Later, each library was amplified by PCR with specific primers for P5 and P7 adapter of Illumina, to increase the molecule number for high-throughput sequencing. Each library was analyzed by Bioanalyzer 2100 system using a DNA High Sensitivity chip. Finally, libraries were quantified fluorometrically using a Qubit®fluorometer with Qubit dsDNA HS Assay kit (Invitrogen). Libraries were amplified clonally into a Flowcell and sequenced on Illumina NextSeq 500 employing a NextSeq 500/550 High Output v2 kit for 150 cycles. Sequencing configuration was indexed Paired-End 2 × 76, following the protocol described by the manufacturer.

### Differential expression analysis by pairwise comparison of strains WG-W3110, WGM-WG, WGMC-WGM, and WHIC-WGMC

RNA-seq reads were quantified by Salmon using default parameters [34], with the *E. coli* K-12 substr. W3110 genome cds file as a reference (ftp://ftp.ncbi.nlm.nih.gov/genomes/all/GCF/000/010/245/GCF_000010245.2_ASM1024v1/GCF_000010245.2_ASM1024v1_cds_from_genomic.fna.gz).

Differential expression analysis was carried out employing edgeR v 3.26.8 [35]. We used the transcript levels from the strain with the higher *qs* value as a reference among pairs. Thus, we performed the following comparisons WG-W3110, WGM-WG, WGMC-WGM, and WHIC-WGMC. In addition, we used the Benjamini-Hochberg (BH) as a *p*-value adjustment method [36]. Differentially expressed genes were those with a *p*-value < 0.01 and False Discovery Rate (FDR) < 0.01 and a fold-change ≥ 2 as the cutoff. We obtained a list of differentially expressed genes for each mutant strain against its respective partner. Afterward, we converted the RefSeq identifiers in Uniprot database (http://www.uniprot.org) using the Retrieve/ID mapping tool to obtain genes names, descriptions, and accession numbers of bnumbers and additional protein information. In addition, we calculated the Pearson correlation coefficient of the differentially expressed genes using their logarithm of fold change (logFC) and their *qs* values. Also, we calculated the slope with the parameter logFC/*qs*, similar to the method reported by Veit et al. 2007 [37].

### COG, annotation of differentially expressed genes

We performed annotation of our list of genes through Cluster of Orthologous Groups (COG’s) using eggNOG-mapper (http://eggnogdb.embl.de/) with DIAMOND mapping mode, the taxonomic scope of Gamma-proteobacteria, prioritizing precision on orthologues, and prioritizing quality on gene ontology evidence. To visualize the results, we used the ggplot2 library based on R.

### RNA extraction, cDNA synthesis and RT-qPCR conditions

To confirm RNA-seq and differential expression analysis results, we performed three RNA extractions and purifications from three independent fermentations for each strain. For this purpose, 50 mL samples were taken of each different strain growing logarithmically. Those samples were collected when the biomass reached 1.0 (OD_600nm_). Then, 1 mL of RNA later buffer (Ambion Inc., USA) was added to each sample, mixed, and centrifuged for 10 min at 4 °C and 5000 rpm. Conditions for total RNA extraction, cDNA synthesis, and RT-qPCR conditions were those previously reported [38], primers used are listed in Additional file 3: Table S1.

## Results

### Growth kinetics characterization

The isogenic mutant strains employed in this study are derived from W3110 and harbor deletions in genes that encode proteins related to carbohydrate import of PTS and non-PTS systems. They belong to a group of strains that displayed a wide range of *qs* values when grown in a minimal medium with glucose [11]. From this group of mutants, we selected five strains that span the full range of observed *qs* values. Strain WG lacks the gene encoding the IICB^Glc^ PTS component. Strain WGM lacks both the IICB^Glc^ and IICD^Man^ components. Whereas strain WGMC lacks the IICB^Glc^, IICD^Man^ components, and the MglABC system. In strain WHIC, genes *ptsHIcrr* were deleted, causing the complete disruption of the PTS system (Fig. 1). Since the PTS phosphorelay chain is disrupted, none of the PTS components is expected to be functional. This strain also lacks the MglABC system.

**Fig. 2:**
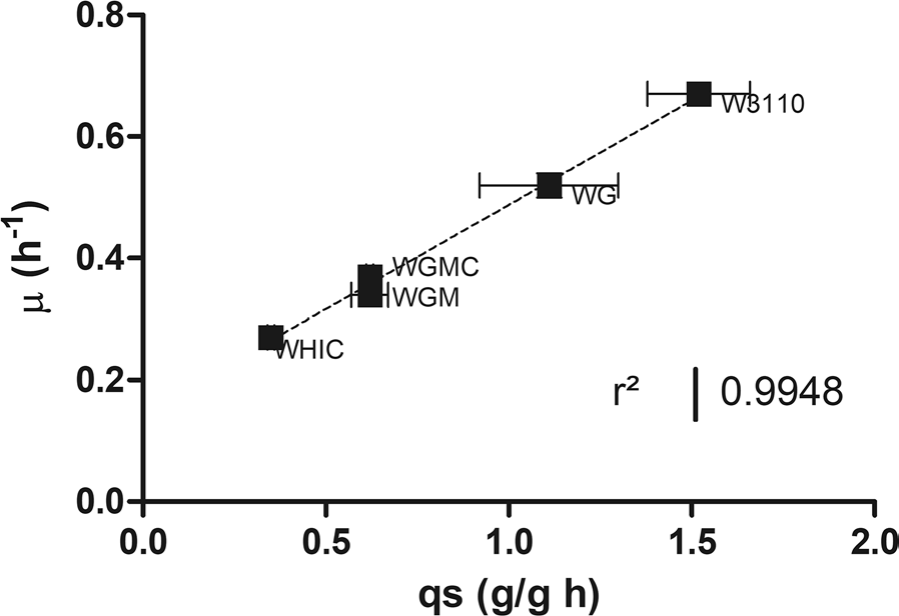
Specific growth rate as a function of specific glucose uptake rate for the *E. coli* strains characterized in this study. Specific growth rate (µ), specific glucose consumption rate (*qs*)

The mutant strains and *E. coli* W3110 were grown in a stirred bioreactor employing minimal salts medium containing 20 g/L glucose. The kinetic and stoichiometric parameters are summarized in Table 2. These strains displayed *qs* and *µ* values that span from 1.52 to 0.35 g/g h and 0.67 to 0.27 h^−1^, respectively (Additional file 1: Fig S1). We observed that *qs* and *µ* values displayed a linear correlation (R_2_ = 0.99; Fig. 2). The final biomass concentration was similar for all strains. The cultures were stopped when the stationary phase was reached at the times listed in Table 2. At these times, we did not detect residual glucose in the medium. Strains W3110, WG, WGMC, and WHIC secreted acetate, reaching the maximum concentration when glucose was depleted, afterwards, the organic acid was consumed. No acetate was detected in the culture medium of strain WGM. Strain W3110 displayed the highest specific rate of acetate production (*q*_*ac*_) and acetate titer of 0.43 g/g h and 1.94 g/L, respectively. The *q*_*ac*_ in W3110 was almost 28-fold higher when compared with the other strains. In these experiments, the biomass yield (Y_X/S_) values differed by less than 20% among all the strains employed in this study.

**Table 2:** kinetic and stoichiometric parameter of cultures with *E. coli* W3110 and derived glucose transport mutants in stirred tank bioreactors

Strain	*µ*(h^−1^)	*qs* (g/g h)	*q*_*ac*_ (g/g h)	Max acetate (g/L)	Y X/S (g/g)	Max biomass (g/L)	Culture time (h)
W3110	0.67 ± 0.03	1.52 ± 0.14	0.433 ± 0.005	1.94 ± 0.04	0.43 ± 0.03	8.4 ± 1.21	10
WG	0.52 ± 0.02	1.11 ± 0.19	0.004 ± 0.001	0.13 ± 0.04	0.41 ± 0.01	8.5 ± 0.23	14
WGM	0.34 ± 0.01	0.62 ± 0.05	ND	ND	0.50 ± 0.02	9.63 ± 0.01	20
WGMC	0.37 ± 0.00	0.62 ± 0.01	0.015 ± 0.001	0.32 ± 0.02	0.44 ± 0.02	7.14 ± 0.64	18
WHIC	0.27 ± 0.01	0.35 ± 0.01	0.001 ± 0.001	0.05 ± 0.03	0.46 ± 0.02	9.23 ± 0.53	28

### Differential expression analysis, reads abundance and mapping

RNA-seq libraries were generated from triplicate samples of WG, WGM, WGMC, WHIC, and W3110 strains. Libraries were sequenced, and 37–48 million reads were obtained from the W3110 library, 31–59, 32–53, 35–56, and 52–54, million reads for WG, WGM, WGMC, and WHIC, respectively. These results indicated that we have generated a similar amount of data among libraries of the strains. Afterward, we used the Salmon program to quantify reads against the annotated *E. coli* K-12 substr. W3110 genome. We obtained on average 3.3 ± 1.2, 2.4 ± 1.2, 3.0 ± 1.1, 4.2 ± 2.1, and 2.6 ± 0.3 million of mapped reads from W3110, WG, WGM, WGMC, and WHIC, respectively, those data are shown in Table 3. The RNA-seq data for this study have been deposited in the European Nucleotide Archive (ENA) at EMBL-EBI under accession number PRJEB32273 (https://www.ebi.ac.uk/ena/browser/view/PRJEB32273).

**Table 3:** Statistics of RNA-seq libraries

Strain	Library size (million reads)	Average library size (million reads)	Mapped reads (million reads)	Average mapped reads (million reads)
W3110	48,37,42	43 ± 5.7	4.7,2.9,2.3	3.3 ± 1.2
WG	59,31,33	41 ± 15.9	4.2,1.4,1.7	2.4 ± 1.2
WGM	32,52,53	46 ± 11.7	2.2,4.2,2.5	3.0 ± 1.1
WGMC	51,56,35	47 ± 11.2	4.7,5.9,1.8	4.2 ± 2.1
WHIC	52,54,54	53 ± 1.5	2.9,2.7,2.3	2.6 ± 0.3

### The global transcriptomic response among strains with a reduced glucose uptake rate

**Fig. 3:**
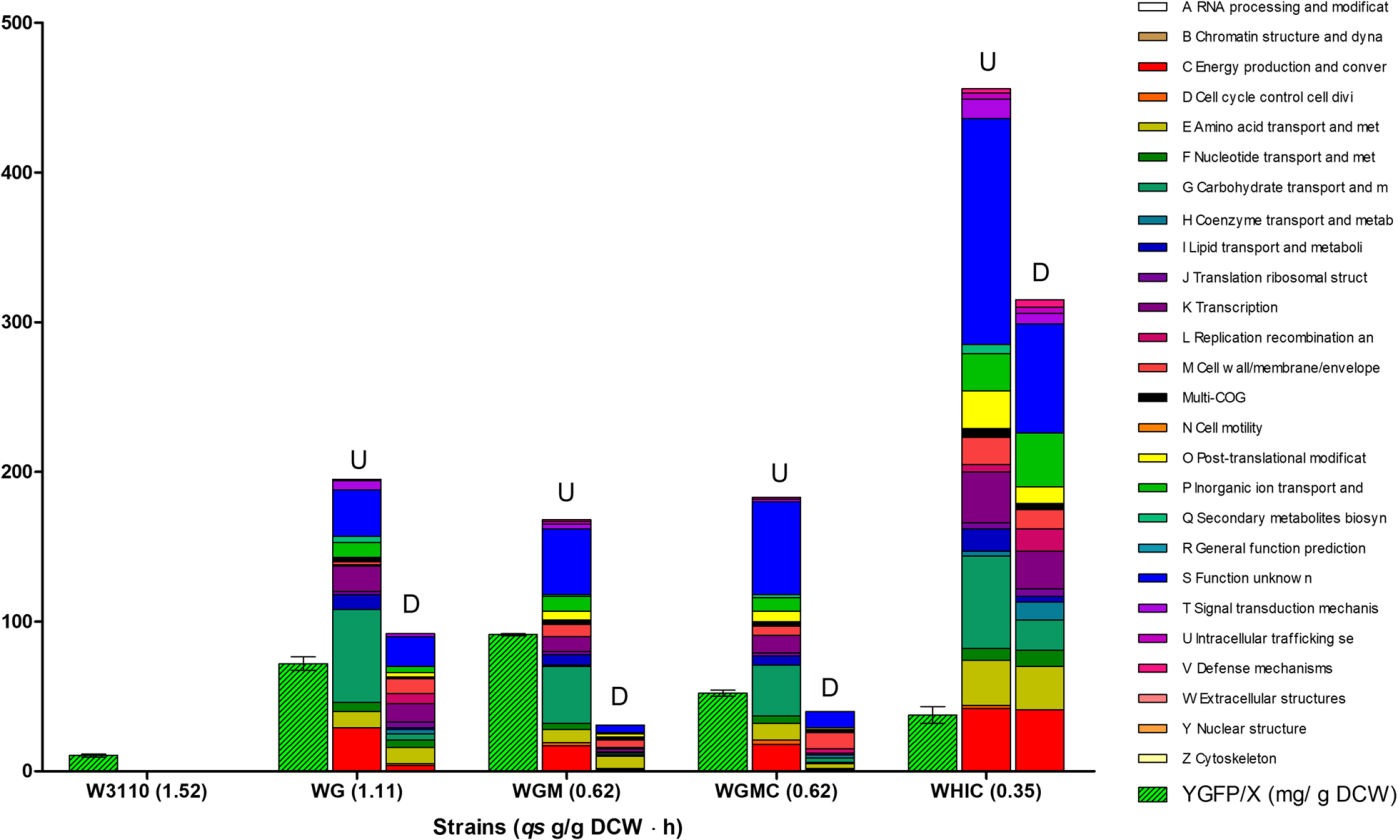
Distribution of differentially expressed genes among mutant strains in Clusters of Orthologous genes when comparing transcriptomes of W3110 with mutant strains WG, WGM, WGMC, and WHIC. The *qs* value for each strain is shown between parenthesis and it is presented as g glucose/g DCW h Values for GFP yield from biomass (Y_GFP/X_) were obtained from a previous publication [11] and are expressed as mg GFP/g DCW

Strains W3110, WG, WGM, WGMC, and WHIC were grown in a minimal medium containing 20 g/L glucose, and samples from these cultures were subjected to RNA-seq analysis. The transcriptomes of the mutant strains were compared to that of W3110. Figure 3 shows a comparison of the number of genes that were differentially expressed when compared to W3110, corresponding to 281, 201, 222, and 779 for strains WG, WGM, WGMC, and WHIC, respectively (Additional file 4: Table S2). It can be observed that in all mutant strains, the number of upregulated genes is higher than those downregulated. The differentially expressed genes belonged to several COGs, as will be discussed below.

**Fig. 4:**
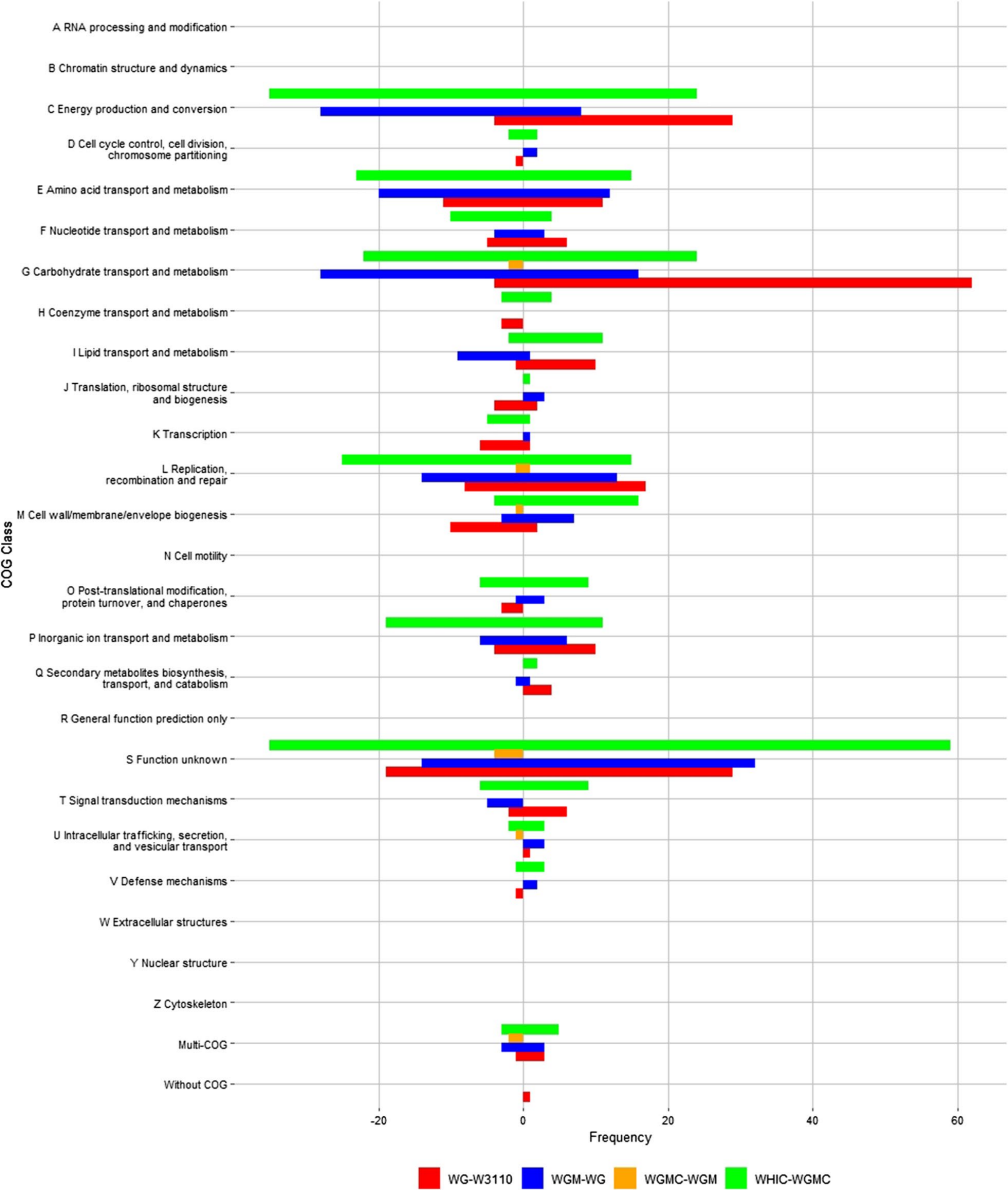
Distribution of differentially expressed genes among pairs of strains in Clusters of Orthologous genes. The number of genes upregulated (positive value) or downregulated (negative value) from each COG is indicated. Transcriptome comparisons: WG-W3110 (yellow), WGM-WG (blue), WGMC-WGM (orange) and WHIC-WGMC (red)

To determine the effects of the change in *qs* (Δ*qs*) among strains, differential expression analysis was performed by comparing pairs of strains with progressively lower *qs* values: W3110-WG (Δ*qs* = 0.41 g/g h), WG-WGM (Δ*qs* = 0.49 g/g h), WGM-WGMC (Δ*qs* = 0 g/g h), and WGMC-WHIC (Δ*qs* = 0.27 g/g h) (Table 4). When performing this comparison, it can be observed that genes clustered in COG S (Function unknown) were the most numerous (Table 5 and Fig. 4). It cannot be discarded that the genes included in this COG have a function related to the production performance of these strains. However, since the functions of these genes are not known, they were not further considered in this study. Most of the genes with known functions belonged to COGs C, E, G, and K, related to energy production and conversion, amino acid transport and metabolism, carbohydrate transport and metabolism, and transcription. Table 4 shows the number of differentially expressed genes for each pairwise comparison. It can be observed that the magnitude of this number shows a partial correlation with the Δ*qs*. An exception to this behavior stands for strains WHIC-WGMC, with a higher-than-expected number of differentially expressed genes to the observed Δ*qs*. This can be explained considering that operon *ptsHIcrr* was deleted in strain WHIC, resulting in the complete disruption of PTS activity. Inactivation of PTS not only eliminates several carbohydrate import functions but also impairs global regulatory functions [39, 40].

**Table 4:** Number of differentially expressed genes in pair-wise comparisons

Strains comparison Counts and direction	WG-W3110	WGM-WG	WGMC-WGM	WHIC-WGMC	WGM-W3110	WGMC-W3110	WHIC-W3110
Up	194	116	1	218	170	182	459
Down	88	136	11	203	31	40	320
Total	281	252	12	421	201	222	779
Δ*qs*	0.41	0.49	0	0.27	0.9	0.9	1.17

**Table 5:** Statistics of differentially expressed genes in Cluster of Orthologous Genes*

Comparison		WG-W3110			WGM-WG			WGMC-WGM			WHIC-WGMC		
COG		U	D	**T**	U	D	**T**	U	D	**T**	U	D	**T**
A	RNA processing and modification	0	0	**0**	0	0	**0**	0	0	**0**	0	0	**0**
B	Chromatin structure and dynamics	0	0	**0**	0	0	**0**	0	0	**0**	0	0	**0**
C	Energy production and conversion	29	4	**33**	8	28	**36**	0	0	**0**	24	35	**59**
D	Cell cycle control, cell division, chromosome partitioning	0	1	**1**	2	0	**2**	0	0	**0**	2	2	**4**
E	Amino acid transport and metabolism	11	11	**22**	12	20	**32**	0	0	**0**	15	23	**38**
F	Nucleotide transport and metabolism	6	5	**11**	3	4	**7**	0	0	**0**	4	10	**14**
G	Carbohydrate transport and metabolism	62	4	**66**	16	28	**44**	0	2	**2**	24	22	**46**
H	Coenzyme transport and metabolism	0	3	**3**	0	0	**0**	0	0	**0**	4	3	**7**
I	Lipid transport and metabolism	10	1	**11**	1	9	**10**	0	0	**0**	11	2	**13**
J	Translation, ribosomal structure and biogenesis	2	4	**6**	3	0	**3**	0	0	**0**	1	0	**1**
K	Transcription	17	8	**25**	13	14	**27**	1	1	**2**	15	25	**40**
L	Replication, recombination and repair	1	6	**7**	1	0	**2**	0	0	**0**	1	5	**6**
M	Cell wall/membrane/envelope biogenesis	2	10	**12**	7	3	**10**	0	1	**1**	16	4	**20**
N	Cell motility	0	0	**0**	0	0	**0**	0	0	**0**	0	0	**0**
O	Post-translational modification, protein turnover, and chaperones	0	3	**3**	3	1	**4**	0	0	**0**	9	6	**15**
P	Inorganic ion transport and metabolism	10	4	**14**	6	6	**12**	0	0	**0**	11	19	**30**
Q	Secondary metabolites biosynthesis, transport, and catabolism	4	0	**4**	1	1	**2**	0	0	**0**	2	0	**2**
R	General function prediction only	0	0	**0**	0	0	**0**	0	0	**0**	0	0	**0**
S	Function unknown	29	19	**48**	32	14	**46**	0	4	**4**	59	35	**94**
T	Signal transduction mechanisms	6	2	**8**	0	5	**5**	0	0	**0**	9	6	**15**
U	Intracellular trafficking, secretion, and vesicular transport	1	0	**1**	3	0	**3**	0	1	**1**	3	2	**5**
V	Defense mechanisms	0	1	**1**	2	0	**2**	0	0	**0**	3	1	**4**
W	Extracellular structures	0	0	**0**	0	0	**0**	0	0	**0**	0	0	**0**
Y	Nuclear structure	0	0	**0**	0	0	**0**	0	0	**0**	0	0	**0**
Z	Cytoskeleton	0	0	**0**	0	0	**0**	0	0	**0**	0	0	**0**
^a^	Multi-COG	3	1	**4**	3	3	**6**	0	2	**2**	5	3	**8**
-	Without COG	1	0	**1**	0	0	**0**	0	0	**0**	0	0	**0**

Comparison		WGM-W3110			WGMC-W3110			WHIC-W3110		
COG		U	D	**T**	U	D	**T**	U	D	**T**
A	RNA processing and modification	0	0	**0**	0	0	**0**	0	0	**0**
B	Chromatin structure and dynamics	0	0	**0**	0	0	**0**	0	0	**0**
C	Energy production and conversion	17	1	**18**	18	1	**19**	42	41	**83**
D	Cell cycle control, cell division, chromosome partitioning	2	1	**3**	3	1	**4**	2	0	**2**
E	Amino acid transport and metabolism	9	8	**17**	11	3	**14**	30	29	**59**
F	Nucleotide transport and metabolism	4	1	**5**	5	1	**6**	8	11	**19**
G	Carbohydrate transport and metabolism	38	1	**39**	34	3	**37**	62	20	**82**
H	Coenzyme transport and metabolism	1	1	**2**	0	2	**2**	3	12	**15**
I	Lipid transport and metabolism	7	0	**7**	6	0	**6**	15	4	**19**
J	Translation, ribosomal structure and biogenesis	2	0	**2**	2	0	**2**	4	5	**9**
K	Transcription	10	2	**12**	12	1	**13**	34	25	**59**
L	Replication, recombination and repair	0	1	**1**	0	3	**3**	5	15	**20**
M	Cell wall/membrane/envelope biogenesis	8	5	**13**	6	11	**17**	18	13	**31**
N	Cell motility	0	0	**0**	0	0	**0**	0	0	**0**
O	Post-translational modification, protein turnover, and chaperones	6	2	**8**	7	1	**8**	25	11	**36**
P	Inorganic ion transport and metabolism	10	1	**11**	9	0	**9**	25	36	**61**
Q	Secondary metabolites biosynthesis, transport, and catabolism	1	0	**1**	2	0	**2**	6	0	**6**
R	General function prediction only	0	0	**0**	0	0	**0**	0	0	**0**
S	Function unknown	44	5	**49**	62	11	**73**	151	73	**224**
T	Signal transduction mechanisms	3	0	**3**	2	0	**2**	13	7	**20**
U	Intracellular trafficking, secretion, and vesicular transport	2	0	**2**	0	0	**0**	4	4	**8**
V	Defense mechanisms	1	0	**1**	1	0	**1**	3	5	**8**
W	Extracellular structures	0	0	**0**	0	0	**0**	0	0	**0**
Y	Nuclear structure	0	0	**0**	0	0	**0**	0	0	**0**
Z	Cytoskeleton	0	0	**0**	0	0	**0**	0	0	**0**
^a^	Multi-COG	3	2	**5**	0	2	**2**	6	4	**10**
-	Without COG	2	0	**2**	2	0	**2**	3	5	**8**

^a^*U* upregulated, *D* downregulated, *T* total

**Fig. 5:**
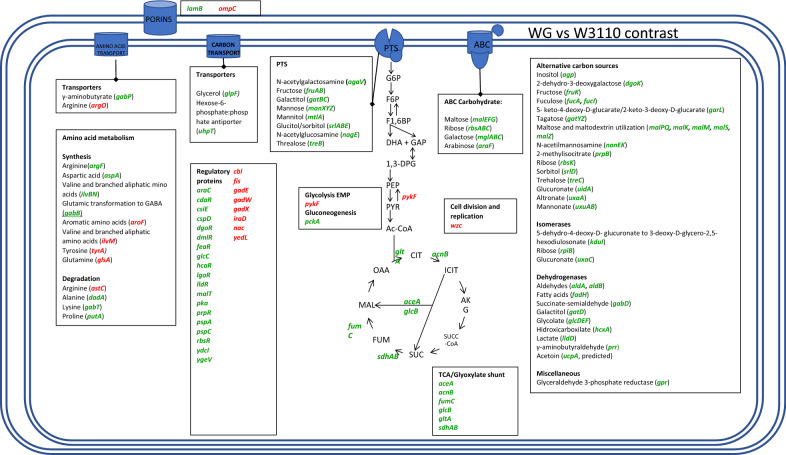
Graphical representation of genes showing differential transcript levels when comparing strains WG and W3110

When comparing the transcriptomes of strains WG and W3110 (Additional file 5: Table S3, Fig. 5), we detected upregulated genes coding for PTS transport and catabolic proteins for N-acetylgalactosamine (*agaV*, 4.3-fold), fructose (*fruABK*, 9.1, 13.8, 12.4-fold), galactitol (*gatBC*, 4.2 and 4.5-fold), mannose (*manXYZ*, 4.3, 3.7, and 4.2-fold), mannitol (*mtlA*, 2.4-fold), glucitol/sorbitol (*srlABED*, 6.3, 7.8, 7.3, 5.7-fold), N-acetylglucosamine (*nagBE*, 2 and 4.2-fold), and trehalose (*treBC*, 19.9 and 18.6-fold) (Fig. 4). Upregulated genes related to non-PTS carbohydrate transport and catabolic proteins were detected for maltose (*malEFG*, *malK,* and *malM*; 8.8–18.1-fold), ribose (*rbsABCK*, and *rpiB*, 13.6, 4.2, 12.7, 3.9, and 4.3-fold), galactose/glucose (*mglABC*, 7.2, 22, and 5.8-fold), arabinose (*araF*, 2.6-fold), inositol (*agp*, 3.5-fold), 2-dehydro-3-deoxygalactose (*dgoK*, 2.1-fold), fuculose (*fucA* and *fucI*, 3.3 and 3.1-fold), 5- keto-4-deoxy-D-glucarate and 2-keto-3-deoxy-D-glucarate (*garL*, 2.7-fold), tagatose (*gatYZ*, 5.4 and fourfold), maltose and maltodextrin (*malPQ, malS,* and *malZ;* 4.7, 3.9, 5.7, and twofold), N-acetilmannosamine (*nanEK*, 3.6 and 2.2-fold), 2-methylisocitrate (*prpB*, 5.1-fold), glucuronate (*uidA* and *uxaC*, 2.3 and 2.4-fold), altronate (*uxaA*, 3.1-fold), mannonate (*uxuAB*¸ 3.4 and 2.5-fold) and 5-dehydro-4-deoxy-D-glucuronate (*kduI*, 2.6-fold). It is noteworthy that none of these PTS and non-PTS carbon sources are present in the culture medium. It is also worth noting that transcriptome data detected, in most cases, the expected coordinated expression of genes when they are part of operons. Genes *acs* (17.4-fold) and *actP* (18-fold) form an operon, and they were found upregulated. The *acs*-*actP* operon encodes proteins of an acetate scavenging pathway, possibly contributing to lowering the accumulation of this organic acid in the culture medium. Also detected, were upregulated genes encoding transport proteins for glycerol (*glpF*, 5.6-fold), the outer porin encoded by *lamB* (14.9-fold) and the hexose-6-phosphate:phosphate antiporter (*uhpT*, 3.5-fold). Another upregulated gene was *glk* (twofold). The transport proteins encoded by the *mglABC* operon and *lamB* constitute a high-affinity pathway for glucose import that is independent of PTS activity. The induction of these genes indicates a carbon source limitation state [13]. The upregulation of *glk* encoding the enzyme glucokinase suggests its participation in the phosphorylation of glucose molecules that are not internalized and phosphorylated by PTS. In strain WG, a lower transcript level of gene *pykF* (-2.1-fold) was detected, it encodes pyruvate kinase 1 from the Embden Meyerhof-Parnas (EMP) pathway. This enzyme is involved in pyruvate formation, so the downregulation of *pykF* suggests diminished glycolytic flux in WG when compared to W3110, resulting in the observed reduced overflow metabolism. Upregulated genes also included part of the glyoxylate and TCA cycles (*aceA*, 3.1-fold; *acnB*, 2.1-fold; *fumC*, 2.3-fold; *glcB*, 4.2-fold; *gltA*, 2.6-fold; and *sdhAB*, 2.4 and 2.2-fold), as well as genes *maeB*¸(twofold) and *pckA* (2.9-fold), that are involved in gluconeogenesis. The induction of gene *pka* (threefold) that is related to the acetylation of proteins was detected in WG. It has been determined that deletion of *pka* leads to lower resistance to heat and oxidative stresses [41].

**Fig. 6:**
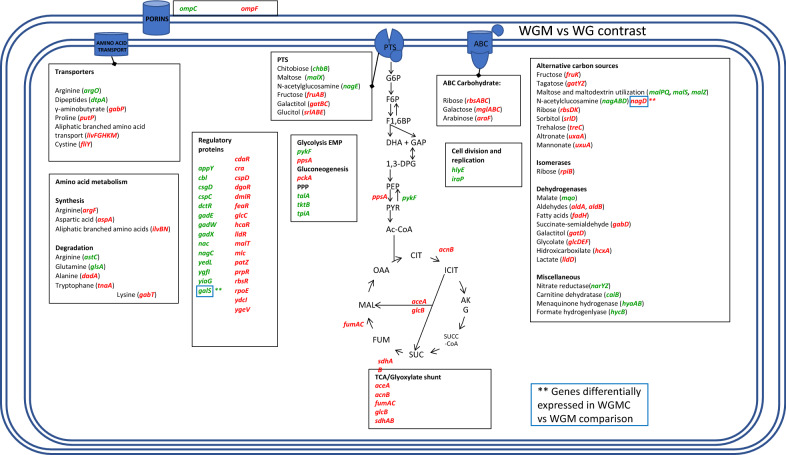
Graphical representation of genes showing differential transcript levels when comparing strains WGM and WG

Comparison of the transcriptomes from strains WGM and WG, showed a further increase in transcript levels in the former strain for genes encoding transport and catabolic proteins for the following carbon sources: chitobiose, trehalose, maltose, and N-acetylglucosamine. The opposite response was observed for transport and catabolic genes for fructose, galactitol, glucitol, arabinose, glycerol, ribose, galactose, trehalose, altronate, and mannonate (Additional file 5: Table S3, Fig. 6). A higher transcript level in WGM was detected for genes *pykF* (2.5-fold), *talA* (2.3-fold), and *tktB* (2.2-fold), indicating a potential for higher flux in the EMP and PP pathways (Additional file 5: Table S3, Fig. 6). Compared to WG, in strain WGM downregulation was observed for genes from the TCA and glyoxylate shunt *gltA* (− 2.7-fold), *aceA* (− 2.5-fold), *acnB* (− 3.1-fold), *fumAC* (− 2.7 and − 2.5-fold), *sdhAB* (− 2.5 and − 2.2-fold), and *glcB* (− 3.1-fold). These data suggest that energy and reducing power production capacity through TCA could be lower in WGM when compared to WG. It is noteworthy that genes involved in gluconeogenesis as well as the PEP/PYS metabolism *ppsA* (− 2.6-fold), *pckA* (-threefold), and the *glpK* (− 3.4-fold) gene which encodes for glycerol kinase were downregulated in this comparison but displayed the opposite response in the WG and W3110 comparison. Upregulation was detected for gene *iraP*, (2.7-fold). The gene *iraP* is required for the stabilization of σS during phosphate or nitrogen starvation and it plays a role in the stationary phase. A higher transcript level for genes *gadX* (4.8-fold) and *gadW* (5.9-fold) was detected in WG. These two genes form a complex operon and encode transcriptional regulators that control the glutamic acid decarboxylase acid resistance system. Genes belonging to this system were found induced: *gadA* (17.3), *gadB* (22.9) *gadC* (26.5), and *gadE* (22.9). It should be noted that pH was controlled and maintained at a neutral value in cultures with these strains. Therefore, the physiological role of this acid resistance system under these conditions is not clear.

Strains WGM and WGMC displayed an identical *qs* value (0.62 g/g h) under the growth conditions employed in this study. This result suggests that the MglABC high-affinity transporter is not contributing to glucose import in strain WGM or that its function was replaced by other proteins in WGMC. Strain WGMC accumulated 0.32 g/L of acetate, while this organic acid was not detected in cultures with WGM. This result indicates that deletion of *mglABC* influences metabolism by an unknown mechanism that causes an increase in acetic acid production [42–44]. The transcriptome analysis yielded 12 differentially expressed genes when comparing WGMC to WGM, most of them without a defined function (Additional file 5: Table S3). Among these genes, *galS* was found upregulated (20-fold), it encodes a transcription factor that regulates the expression of genes involved in the transport and catabolism of D-galactose. However, no increase in transcript level was detected for members of the GalS regulon. Further characterization of WGMC should be useful to determine how the deletion of *mglABC* causes the observed metabolic and transcriptional responses.

**Fig. 7:**
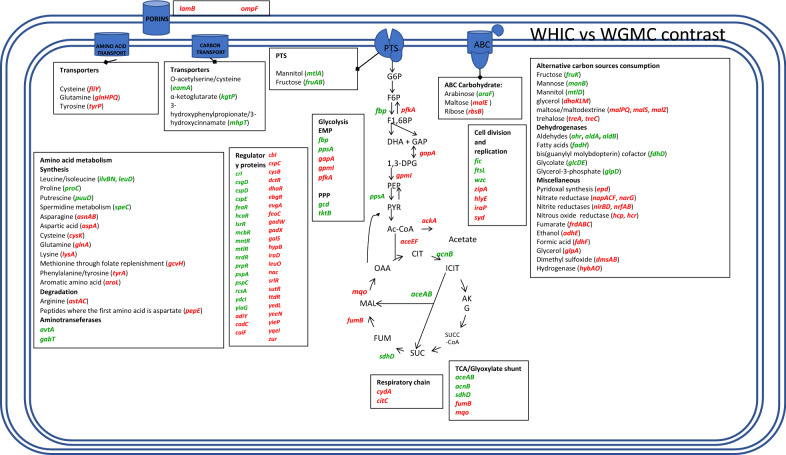
Graphical representation of genes showing differential transcript levels when comparing strains WHIC and WGMC

The transcriptome comparison of WHIC to WGMC yielded upregulated genes encoding transport and/or catabolic proteins for the consumption of fructose, arabinose, O-acetylserine/cysteine, α-ketoglutarate, 3-hydroxyphenylpropionate/3-hydroxycinnamate, glycerol, maltose/maltodextrin, mannose, ribose, and trehalose (Additional file 5: Table S3, Fig. 7). In contrast to the response observed in mutant strains WG and WGM, gene *lamB* (-3.1-fold) was downregulated. This result is consistent with the reported poor expression for this gene under glucose excess and glucose-starved conditions [13]. A higher transcript level was detected for gluconeogenic genes *fbp* (2.1-fold) and *ppsA* (3.9-fold) (Additional file 5: Table S3, Fig. 7). In contrast, several genes from the EMP pathway were downregulated (*gapA*, − 2.5-fold; *gpmI*, − 3.7-fold; *pfkA*, and − 2.3-fold). Gene *cydA* (− 2.8-fold), which is related to the respiratory chain, was downregulated. Also detected were lower transcript levels of genes encoding part of the pyruvate dehydrogenase (*aceEF*, 3.2 and − 2.6-fold), and *ackA* (− 2.9-fold) related to acetic acid assimilation/production. Genes related to the synthesis of glutamine, asparagine cysteine, and lysine (*glnA* − 7.7-fold, *asnAB* − 5.6 and − 5.9-fold, *cysK* − 3.2-fold, *lysA* -twofold) were downregulated. A lower transcript level was observed for gene *iraP* (− 2.8-fold). In this case, the response of this gene was opposite to that observed for the WG-W3110 and WGM-WG transcriptome comparisons. Genes encoding functions related to the synthesis of the extracellular polysaccharide colonic acid displayed higher transcript level in strain WHIC: *wcaA* (3.9-fold), *wcaC* (3.2-fold), *wcaD* (4.5-fold), *wcaE* (6.6-fold), *wcaF* (8.7-fold), *wcaI* (fourfold), *wcaJ* (fourfold), *wza* (7.7-fold), *wzb* (7.4-fold), and *wzc* (3.2-fold). Strain WHIC displayed the lowest *qs* among studied mutants and the observed transcriptome response is characteristic of severe carbon and energy limitation. As will be discussed below, part of the observed response can be explained considering the dual transport-regulatory role of PTS in *E. coli*.

The set of differentially expressed genes and their intersections in the WG-W3110, WGM-WG, and WHIC-WGMC pairwise comparisons are shown in Additional file 1: Fig S2 and Additional file 6: Table S4. These comparisons were tagged as A, B, and C, in the Venn diagram respectively. It is important to mention that we did not include the WGMC-WGM comparison because they displayed very similar *µ* and *qs* values (see Additional file 6: Table 4) and a nearly identical transcriptomic response. Comparisons A, B, and C had unique genes that appeared to be more numerous in WGMC-WHIC (C; 287), followed by WG-W3110 (A; 96) and WGM-WG (B;65).

### Validation of gene expression by qPCR

RT-qPCR experiments were performed for the following genes displaying differential expression in the RNA-seq results: *galP*, *manX*, *nagE*, *lamB*, *ompC*, *ompF*, *mglB*, *gltA*, *acs*, *poxB* and *ppsA*. (Table 6). The magnitude of the expression levels detected by qPCR was different to those observed in the RNA-seq experiments, but the general response regarding upregulation or downregulation was conserved for most of the genes: *manX*, *nagE*, *lamB*, *ompC*, *mglB*, *gltA*, *acs*, *poxB* and *ppsA*.

**Table 6:** qPCR and RNA-seq results of genes from carbon transport systems and central metabolism

	Gene	RT-qPCR^a^								RNA-seq			
		WG		WGM		WGMC		WHIC		WG	WGM	WGMC	WHIC
		AVG	SD	AVG	SD	AVG	SD	AVG	SD				
Transport systems	*galP*	1.19	0.01	1.63	0.29	1.48	0.18	1.59	0.37	− 0.43	0.18	0.58	1.40
	*manX*	5.88	1.25	ND	ND	ND	ND	1.19	0	2.11	ND	ND	1.80
	*nagE*	4.38	0.54	43.34	4.43	53.34	3.83	30.95	11.49	2.08	5.10	5.04	5.96
	*lamB*	22.73	4.61	8.42	0.18	7.27	0.45	1.64	0.2	3.89	3.69	3.86	2.25
	*ompC*	0.25	0.11	0.73	0.06	0.91	0.27	0.96	0.14	− 1.00	0.46	0.5	0.55
	*ompF*	2.93	0.07	0.58	0	0.56	0.08	0.14	0.02	0.97	− 0.61	− 0.26	− 1.55
	*mglB*	29.11	10.46	4.55	0.62	ND	ND	ND	ND	4.46	1.88	ND	ND

	Gene	RT-qPCR								RNA-seq			
		WG		WGM		WGMC		WHIC		WG	WGM	WGMC	WHIC
		AVG	SD	AVG	SD	AVG	SD	AVG	SD				
TCA	*gltA*	3.2	1	0.65	0.02	0.93	0.05	1.39	0.24	1.35	− 0.10	0.20	0.80

	Gene	RT-qPCR								RNA-seq			
		WG		WGM		WGMC		WHIC		WG	WGM	WGMC	WHIC
		AVG	SD	AVG	SD	AVG	SD	AVG	SD				
Acetate	*acs*	27.58	4.97	3.42	0.39	3.83	0.32	3.13	0.44	4.12	1.74	1.84	2.61
	*poxB*	0.44	0.02	1.78	0.23	1.84	0.37	1.55	0.2	0.38	1.16	1.21	1.98
	*ppsA*	1.82	0.37	0.41	0.06	1.89	0.53	3.72	0.4	0.98	− 0.39	0.48	2.37

^a^Values represent the logarithm of fold change when comparing gene expression values from genes of each strain against *E. coli* W3110. Dispersion values are not presented from RNA-seq results but can be consulted in the supplementary files

### Correlations between *qs* and expression levels

In this study, we detected genes displaying expression levels that correlated with the *qs*. This response was characterized by calculating the Pearson correlation coefficient between *qs* and the logarithm of fold change (logFC). Also, we defined that (ΔlogFC/Δqs) ratios larger than 1.5 or lower than − 1.5 were considered as differentially expressed among the full set of strains. Briefly, a negative correlation indicates that genes increase their expression values while the *qs* value is decreasing. In contrast, a positive correlation means that genes decrease their expression level while *qs* is decreasing. Lists of genes displaying positive and negative correlations with *qs* are included in Additional file 6, 7: Tables S5 and S6, respectively. The results were then organized in COG classes to facilitate their analysis. Of 292 genes having a positive or negative correlation, 47% are not classified in a COG class. The remaining 138 genes were distributed in the O, P, M, K, C, E, S, and G classes.

Inspection of COG C shows 20 genes displaying a positive correlation with *qs*, among them we found the *nuoFGHIJKLM operon* and the phosphoenolpyruvate carboxylase (*pckA*). Also, dehydrogenases for glycerol (*glpA*), sorbitol (*srlD*), galactitol (*gatD*), hydroxycarboxylate (*hcxA*), and acetoin (*ucpA*). Conversely, we detected 13 genes with negative correlation to *qs*, the pyruvate dehydrogenase (*poxB*), ubiquinol reductase (*appBC*), 2-methylcitrate synthase (*prpC*), and an alcohol/ethanol dehydrogenase (*adhP*). From this set, the response of the *nuo* genes can be explained considering that energy production requirements are lower under the reduced growth rates of the mutant strains. This assumption should be tested experimentally since *E. coli* has a second NADH dehydrogenese encoded by *ndh*. The *nuo* operon forms part of the DNA-binding transcriptional dual regulator Fis regulon, it has been demonstrated that repression by Fis, a growth-dependent regulator, occurs when this transcription factor is in high concentrations [45].

We detected 16 genes from COG E with positive and 9 with negative correlations. Among the former, there were genes related to the synthesis of arginine (*argB* and *argF*), aspartic acid (*aspA*), cysteine (*cysK*), histidine (*hysG*), serine/threonine (*sstT*), proline (*proW*), and cysteine (*fliY*). Decreased expression of genes encoding functions related to amino acid biosynthesis can be expected because of the lower growth rates of the mutant strains with the lower *qs* values. However, it is not clear why this response is selective for some amino acids. Genes displaying a negative correlation with *qs* included *gadA* and *gadBC* that encode proteins of the glutamate-dependent acid resistance system 2. Expression of these genes is increased in the mutant strains displaying the lower *qs* values. Since pH is maintained at a neutral value in these cultures, the upregulation of these genes should be dependent on another type of stress or metabolic signal. It should be noted that these genes are subject to complex regulation by transcriptional factors GadX, GadW, Fis, and CRP [46].

From COG G we found 22 and 19 genes with positive and negative correlations, respectively. Genes showing a positive correlation with *qs* encode functions related to galactitol, glucitol/sorbitol, trehalose PTS transport systems (*gatC*, *srlABC*, and *treB*), the ribose (*rbsBC*) and maltose (*malG*) ABC transporters, as well as a set of genes encoding proteins for the utilization of ribose (*rbsDK*), trehalose (*treC),* fucose (*fucA* and *fucI*), tagatose (*gatZ*), galactonate (*dgoA*), methylglyoxal (*mgsA*), and altronate (*uxaAC* and *uxaB*). These genes are likely induced as a response to carbon limitation in strain WG. Paradoxically, their expression is progressively reduced in strains with lower *qs*, possibly as a result of energy limitation or another type of metabolic perturbation. In contrast, genes encoding PTS transport proteins for chitobiose (*chbB*) and N-acetylglucosamine (*nagE*), and the non-PTS galactose symporter (*galP*) displayed a negative correlation. From these proteins, GalP has been shown to replace the PTS-dependent glucose transport function in a mutant lacking a functional PTS system [47]. Therefore, GalP may be contributing to glucose import in some of these mutants. Interestingly, some mutant strains showed increased expression of *glk*, encoding the enzyme glucokinase that can phosphorylate the glucose internalized by GalP.

Three genes from this COG J class displayed a negative correlation (*argS*, *rmf*, and *sra*). The *argS* gene encodes for the arginyl-tRNa synthetase. The *rmf* encodes for a ribosome modification factor which reversibly converts active 70S ribosomes into their 100S dimeric form during the transition from exponential growth to stationary phase [48]. Gene *sra* encodes for a protein associated to the ribosomal 30S subunit during the stationary phase [49]. The observed expression pattern for genes *sra* and *rmf* indicate that mutant strains with the lower *qs* values have a physiological state like that observed in the stationary phase. These results could indicate that strains with lower *qs* values have a physiological state that is not optimal for recombinant protein production.

## Discussion

This study aimed to characterize *E. coli* strains with impaired capacity for glucose import. We expect these data and their analysis will provide an insight into the basis for their distinct microbial factory characteristics [12]. Transcriptome analysis was performed on *E. coli* strain W3110 and isogenic derivatives reduced glucose consumption capacity: WG, WGM, WGMC, and WHIC.

The number of differentially expressed genes when comparing WG-W3110 and WGM-WG was similar, indicating a correlation with the *qs* ratio among each pair of strains (Table 4). In contrast, strains WGMC and WGM displayed nearly identical *qs* values. The only difference between these strains is the deletion of the *mglABC* operon. In this case, 12 genes were found to be differentially expressed. Strain WGMC produced 0.32 g/L of acetic acid, while in the culture medium of WGM we did not detect this organic acid (Table 2). This phenotypic difference could explain the set of differentially expressed genes. However, further strain characterization will have to be performed to verify this assumption. When comparing strains WHIC and WGMC the Δ*qs* was about half of that detected for WG-W3110 and WGM-WG. However, the number of differentially expressed genes was about twice as expected. These results show that transcriptomic response does not have a linear relationship with changes in *qs*. It should be noted that the *qs* value for WHIC is only 23% of that determined for W3110. These data suggest that very low *qs* values cause stress that elicits a large transcriptomic response. It should also be considered that deleted glucose import proteins could have a regulatory role. This is the case for strain WHIC, the deletion of operon *ptsHIcrr* eliminates the synthesis of protein EIIA^Glc^, thus disrupting the stimulation of cAMP synthesis by enzyme adenylate cyclase. Therefore, the expression of genes from the CRP regulon should be disrupted in strain WHIC. In this regard, it was found that in the WHIC-WGMC comparison there were 48 genes regulated by CRP, (33 belonging to COGs C, E, G, and K). This number is lower than that detected when comparing WG-W3110, 76 genes (53 from COGs C, E, G, and K) and WGM-WG 56 genes (39 from COGs C, E, G, and K). The transcription factor CRP-cAMP regulates several hundred genes in *E. coli*, and some of them are also transcriptional regulators [29]. The obtained data suggest that the observed transcriptomic response in WHIC is the combined effect of very low *qs*, which would induce many genes related to carbon source limitation, and the indirect disruption of the CRP regulon.

Strain WG is isogenic with W3110 except for the deletion of the gene encoding IICB^Glc^ in the former. This genetic modification caused a transcriptional response consistent with a carbon limitation and hunger response characterized by a decrease in transcript level for genes encoding functions related to glycolytic activity and an increase in expression of genes from gluconeogenic metabolism. The expected reduction in overflow metabolism was observed in cultures with this strain with 6.7% of the acetate titer detected in cultures with W3110 (Table 2). As part of the hunger response, genes involved in the transport and utilization of several different carbohydrates and amino acids were upregulated. It should be noted that these compounds were not present in the culture medium, so the observed response should be a carbon source scavenging strategy. Additionally, several genes related to amino acid synthesis were downregulated, possibly caused by the 22% lower growth rate of WG when compared to W3110, resulting in lower demand for amino acids.

The deletion of *manX* in the *ptsG* background results in strain WGM that lacks two PTS components related to glucose import [11]. Strain WGM lacks gene *manX*, so PTS components IIA^man^ and IIB^man^ are not synthesized. This strain displays *µ* and *qs* values corresponding to 65 and 56% of those observed for WG (Table 2). Acetate was not detected in cultures with WGM. The transcriptome data showed that the further decrease in glucose import capacity in strain WGM, when compared to WG, caused the induction of additional genes encoding PTS and non-PTS proteins involved in the uptake and catabolism of secondary carbon sources that were not present in the culture medium. Interestingly, the observed transcriptional response included several genes from the EMP and PPP that were found upregulated. A lower level of transcripts was detected for genes from the TCA cycle. This response suggests a decrease in energy production capacity for WGM when compared to WG, likely because of the lower growth rate. Strain WGM displayed a higher transcript level for gene *iraP* (2.7-fold), encoding a small anti-adaptor protein that is required for stabilization of the alternative sigma factor σ^S^. The expression of *iraP* is dependent on phosphate starvation and to a lesser extent on carbon limitation, and this response is dependent on ppGpp [50]. These data suggest an involvement of σ^S^ in the observed transcriptional response in WGM. The transcriptome data is indicative of a more severe carbon limitation when comparing WGM to WG. It should be noted that no acetate accumulation was detected in cultures with strain WGM, thus, overflow metabolism was eliminated in this strain (Table 2). A derivative of strain WGM that expresses *gfp* displayed a 1.4-fold increase in the specific rate of GFP synthesis and its yield from biomass when compared to isogenic WG. Strain WGM displayed the highest GFP production capacity when compared to production strains derived from all mutants in this study [12]. This improvement in recombinant protein production capacity for WGM when compared to other strains in this study, can be attributed in part to the specific state of carbon source limitation, and the complete elimination of overflow metabolism.

The effect of eliminating the high-affinity glucose uptake system MglABC on the WGM background was studied by comparing it with strain WGMC. This modification did not alter the *qs*. However, acetate was produced to a maximum level of 0.32 g/L, whereas this organic acid was not detected in cultures with strain WGM. Among the 12 genes that showed differential expression in WGMC when compared to WGM, *nagD* was found to be downregulated (− 2.2-fold) and *galS* upregulated (20.1-fold). Gene *nagD*, also known as *umpH* is part of the N-acetylglucosamine utilization operon and encodes a ribonucleotide phosphatase [51]. It has been determined that overexpression of *nagD* results in the induction of genes related to cell envelope and heat shock stress. The co-expression of *nagD* and mammalian G protein-coupled receptors (GPCR) in *E. coli* resulted in a 3- to tenfold increase in the yield of the latter [52]. This result can be explained considering that induction of stress-related proteins provides a favourable environment to produce recombinant proteins. It remains to be determined if the lower transcript level of *nagD* in WGMC could explain in part why this strain displayed lower GFP production capacity when compared to WGM [12]. Gene *galS*, also known as *mglD* encodes the galactose isorepressor, a transcription factor that represses the transcription of genes involved in galactose catabolism [53]. It has been reported that growth under a low glucose concentration causes the synthesis of galactose as an autoinducer of the *gal* regulon [14, 54]. The *gal* regulon contains genes encoding proteins involved in the transport and metabolism of galactose. It should be noted that the transport proteins that are members of this regulon, GalP, and MglABC, can also employ glucose as substrate [11]. When comparing GFP production strains derivatives of WGM and WGMC it was determined that the latter produced about 40% less recombinant protein [12]. This result can be explained considering the increased production of acetate and possibly also the downregulation of *nagD*. However, with the available data, it is not possible to determine how the deletion of *mglABC* caused such effects. It is interesting to note the lack of correlation between the *qs* and *q*_*ac*_ values when comparing strains WGM and WGMC. Elimination of MglABC may abolish the ATP-dependent high-affinity import of glucose that is dependent on ATP for phosphorylation. If MglABC has an important role in glucose import in strain WGM, the deletion of this transporter could shift glucose import to systems dependent on energy from PEP (PTS) or proton motive force (GalP). This modification could alter the energetic state of the cell and central metabolic fluxes, indirectly affecting acetate production in WGMC. These results suggest that deletion of operon *mglABC* should be carefully considered as a strategy to modify *qs* in a production strain since, as shown here, this modification causes unexpected results. Further strain characterization, including measurement of the energy state, as well as carbon flux determination in central pathways should help in understanding the consequences of this modification.

Strain WHIC displayed the lowest values for *µ* and *qs* among studied strains and an extremely low level of acetate production (Table 2). Strain WHIC showed the largest number of differentially transcribed genes when compared to the other strains in this study. Even when comparing transcriptome data of WHIC with the strain showing the closest *qs* value, WGMC, 421 genes were found to be expressed differentially. In this comparison, several genes encoding PTS components as well as genes encoding proteins for the transport and metabolism of alternate carbon sources were upregulated in WHIC. Downregulation of genes from the respiratory chain was detected. In the context of GFP production, this strain displayed a threefold higher titer when compared to an isogenic producer strain derived from W3110 [12]. However, WHIC showed the lowest GFP titer when compared to the other mutant strains in this study. The transcriptome profile shown by WHIC suggests the most severe level of carbon and energy limitation when compared to the other mutant strains. Overflow metabolism is reduced and nearly non-existing. However, the extreme hunger state displayed by this strain can be considered the cause for the relatively low GFP production capacity when grown in minimal salts media.

When compared to the transcriptome of wild-type strainW3110, all isogenic derivatives with reduced glucose consumption capacity displayed a relatively large number of differentially expressed genes. The ratios of upregulated to downregulated genes corresponded to 2.2, 5.5, 4.6, and 1.4 for strains WG, WGM, WGMC, and WHIC, respectively. It is noteworthy that even though these strains displayed a transcriptomic response consisting mainly of upregulated genes, all of them have shown an improved capacity for recombinant protein production when compared to W3110 [12]. The observed response suggests that a strategy for further strain optimization for recombinant protein production could be based on avoiding the upregulation of non-essential functions. This approach, also known as resource allocation engineering, has been proven to be effective in improving microbial cell factories [55]. In the case of the glucose transport mutant strains employed in this study, resource allocation engineering could be employed to determine if avoiding the induction of scavenging functions for carbon sources that are not present in the culture medium and other non-essential functions would improve performance for recombinant protein production. It should be noted that several research groups have reported the improvement of *E. coli* strains to produce recombinant proteins or chemicals by employing mutants with reduced glucose import rates [10, 32, 56]. Those strains may be improved by applying an engineering strategy based on eliminating the non-essential induced functions identified in this study.

## Supplementary Information

**Additional file 1: Figure S1** Growth kinetics of strains W3110, WG, WGM, WGMC, and WHIC. Glucose concentration (triangles), biomass concentration (squares) and acetate concentration (diamonds).

**Additional file 2: Figure S2** Venn diagram representing the number of unique and overlapping genes among strain transcriptome comparisons. Comparisons WG-W3110, WGM-WG, and WHIC-WGMC were tagged as A, B, and C, respectively. The WGMC-WGM comparison was excluded from this analysis.

**Additional file 3:** Supplementary table 1 Primers used in this study.

**Additional file 4:** Supplementary table 2 List of differentially expressed genes when comparing the transcriptome of W3110 with those of the mutant strains.

**Additional file 5:** Supplementary table 3 List of differentially expressed genes when performing pair-wise comparison of transcriptomes from strain in this study.

**Additional file 6:** Supplementary table 5 List of genes displaying a positive correlation of expression with the specific rate of glucose consumption.

**Additional file 7**: ﻿Supplementary table 5 List of genes displaying a negative correlation of expression with the specific rate of glucose consumption.

## Data Availability

The datasets used and/or analyzed during the current study are available from the corresponding authors on reasonable request.

## Abbreviations

AcCoA: Acetyl-coenzyme A
Ack: Acetate kinase
Spc: Spectinomycin
LB: Luria Bertani
CDW: Cell dry weight
GFP: Green fluorescent protein
Pta: Phosphotransacetylase
PEP: Phosphoenolpyruvate
LacI: Lactose repressor
PTS: Phosphoenolpyruvate:sugar phosphotransferase system
TCA: Tricarboxylic acid cycle
q_ac_: Specific rate of acetate production
q_s_: Substrate uptake rate
q_p_: Recombinant product formation rate
SD: Standard deviation
*gfp*: Superglo GFP gene
Y_X/S_: Biomass yield from glucose
