# Supplementary figures and images for: Glucose consumption rate-dependent transcriptome profiling of Escherichia coli provides insight on performance as microbial factories
Source: Microb Cell Fact. 2022 Sep 14;21:189. doi: 10.1186/s12934-022-01909-y (PMC9472385; doi:10.1186/s12934-022-01909-y)

## Slide 1
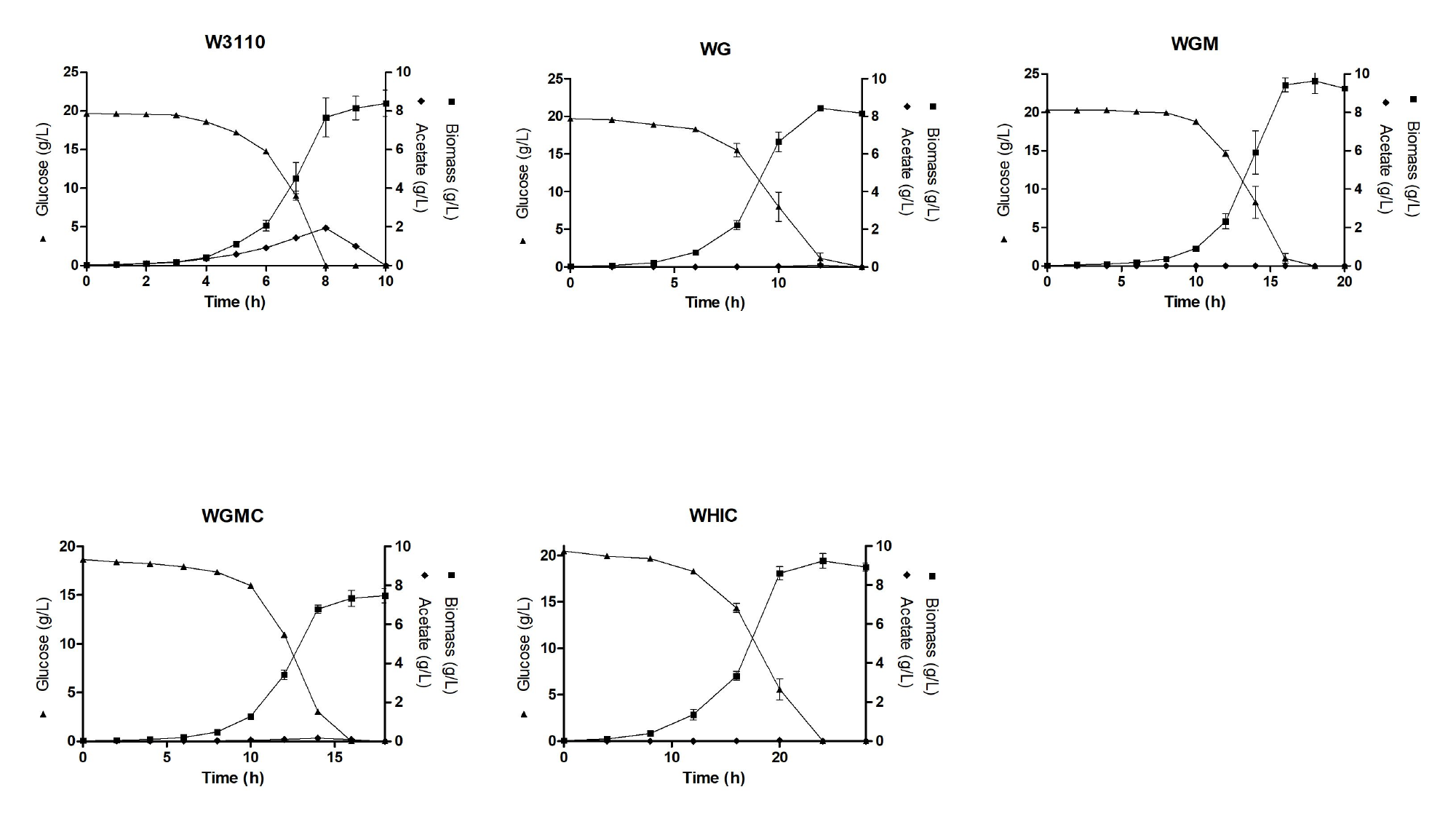

Supplement: Supplementary file 1 — Additional file 1: Figure S1 Growth kinetics of strains W3110, WG, WGM, WGMC, and WHIC. Glucose concentration (triangles), biomass concentration (squares) and acetate concentration (diamonds). [file 12934_2022_1909_MOESM1_ESM.pptx]

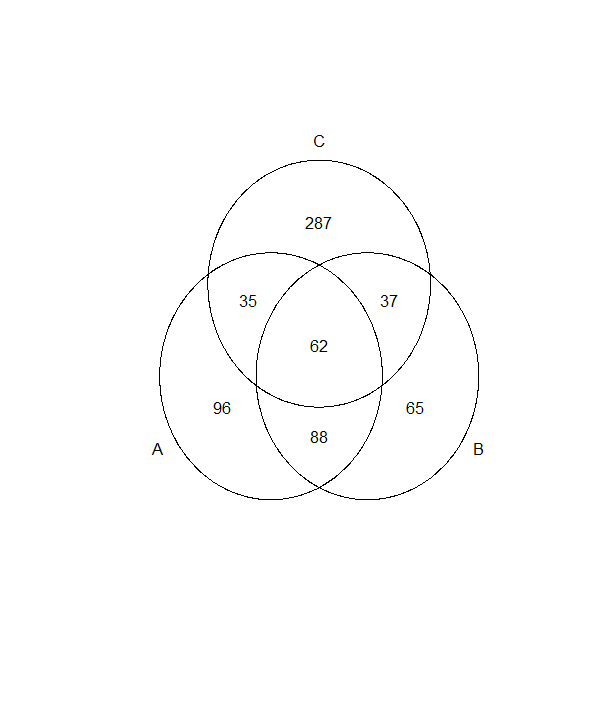

Supplement: Supplementary file 2 — Additional file 2: Figure S2 Venn diagram representing the number of unique and overlapping genes among strain transcriptome comparisons. Comparisons WG-W3110, WGM-WG, and WHIC-WGMC were tagged as A, B, and C, respectively. The WGMC-WGM comparison was excluded from this analysis. [file 12934_2022_1909_MOESM2_ESM.tiff]
