# Supplementary material for: Glucose consumption rate-dependent transcriptome profiling of Escherichia coli provides insight on performance as microbial factories
Source: Microb Cell Fact. 2022 Sep 14;21:189. doi: 10.1186/s12934-022-01909-y (PMC9472385; doi:10.1186/s12934-022-01909-y)
Supplement: Supplementary file 3 — Additional file 3: Supplementary table 1 Primers used in this study. [file 12934_2022_1909_MOESM3_ESM.docx]

| **Suplementary table 1** | | |
| --- | --- | --- |
| **Primers used in this study*** | | |
| **Gene** | **Primer name** | **Sequence (5’— 3’)** |
| *galP* | galPa | CATGTATTACGCGCCGAAAA |
|  | galPb | TGGCAAGTACGTTGGTCAGG |
| *manX* | manXa | TGCAGGCGTTAACATTCCAA |
|  | manXb | CACGGCCTGTTTCTACTGCC |
| *nagE* | nagEa | GGCGGTGAAACCGACAGATA |
|  | nagEb | CTTTTTCGGTTTCCAGGCAG |
| *lamB* | lamBa | AACTTCCTCTGGCGGTTGC |
|  | lamBb | ACCTGTCCAACCAATACCGG |
| *ompC* | ompCa | GCGTCTTGGCTTCAAAGGTG |
|  | ompCb | TGTTTTCGTTTTCAGCGCTG |
| *ompF* | ompFa | TTCGCGGGTCTTAAATACGC |
|  | ompFb | AATTCTGGCAGCATATCGGTG |
| *mglB* | mglBa | CCAGCATGTTATTCGGTGCC |
|  | mglBb | AGCCTTGCGCACTACAGACA |
| *acs* | acsa | GTGCGTAAAGAGATTGGCCC |
|  | acsb | CGCAGAATACGGCGCATAAT |
| *poxB* | poxBa | AAAAGCCGATCGCAAGTTTC |
|  | poxBb | GGTGAATGGCTTTCTCGCTC |
| *ppsA* | ppsa | TCAGCAGGAAACCTTCCTCAA |
|  | ppsb | GATAAGAGATGGCGCGATCG |
| *gltA* | gltAa | AGGCACGCTGGGTCAAGAT |
|  | gltAb | TAGATTCGCAGGATGCGGTT |
|  | | |

*Letters after primers name mean: a, forward primer; b, reverse primer.
